# A novel Silva pattern-based model for precisely predicting recurrence in intermediate-risk cervical adenocarcinoma patients

**DOI:** 10.1186/s12905-022-01971-z

**Published:** 2022-09-16

**Authors:** Chenyan Guo, Xiang Tao, Lihong Zhang, Ying Zhang, Keqin Hua, Junjun Qiu

**Affiliations:** 1 Department of Gynecology, Obstetrics and Gynecology Hospital, Fudan University, 419 Fangxie Road, Shanghai, 200011 China; 2 Shanghai Key Laboratory of Female Reproductive Endocrine-Related Diseases, 413 Zhaozhou Road, Shanghai, 200011 China

**Keywords:** Cervical adenocarcinoma, Recurrence, Prediction model, Silva

## Abstract

**Background:**

Considering the unique biological behavior of cervical adenocarcinoma (AC) compared to squamous cell carcinoma, we now lack a distinct method to assess prognosis for AC patients, especially for intermediate-risk patients. Thus, we sought to establish a Silva-based model to predict recurrence specific for the intermediate-risk AC patients and guide adjuvant therapy.

**Methods:**

345 AC patients were classified according to Silva pattern, their clinicopathological data and survival outcomes were assessed. Among them, 254 patients with only intermediate-risk factors were identified. The significant cutoff values of four factors (tumor size, lymphovascular space invasion (LVSI), depth of stromal invasion (DSI) and Silva pattern) were determined by univariate and multivariate Cox analyses. Subsequently, a series of four-, three- and two-factor Silva-based models were developed via various combinations of the above factors.

**Results:**

(1) We confirmed the prognostic value of Silva pattern using a cohort of 345 AC patients. (2) We established Silva-based models with potential recurrence prediction value in 254 intermediate-risk AC patients, including 12 four-factor models, 30 three-factor models and 16 two-factor models. (3) Notably, the four-factor model, which includes any three of four intermediate-risk factors (Silva C, ≥ 3 cm, DSI > 2/3, and > mild LVSI), exhibited the best recurrence prediction performance and surpassed the Sedlis criteria.

**Conclusions:**

Our study established a Silva-based four-factor model specific for intermediate-risk AC patients, which has superior recurrence prediction performance than Sedlis criteria and may better guide postoperative adjuvant therapy.

**Supplementary Information:**

The online version contains supplementary material available at 10.1186/s12905-022-01971-z.

## Background

As the second most common histologic type of cervical cancer, cervical adenocarcinoma (AC) comprises 10–25% of cervical cancer cases [1]. With the popularization of screening and the promotion of human papillomavirus (HPV) vaccines, the incidence of cervical cancer is decreasing. However, the incidence of AC is on the rise [2]. Unlike squamous cell carcinoma (SCC), AC has different epidemiologies, unique tumor biological behaviors and endogenous characteristics [3, 4]. Studies have shown that AC carries a worse prognosis, with 10–20% differences in 5-year overall survival (OS) rates [5–7], and has a greater risk of developing distant metastases (AC 25%, SCC 14%) than SCC [8]. Thus, there is an urgent need to focus on AC and to establish a unique prognostic estimation and postoperative adjuvant treatment decision-making system for AC patients.

At present, the modality of postoperative adjuvant therapy for AC is based on the presence of several risk factors according to National Comprehensive Cancer Network (NCCN) guidance [9], which is the same as SCC. For patients with any one of the high-risk factors (positive lymph node (LN), parametrial involvement or surgical resection), adjuvant chemoradiation is needed. For those who have positive intermediate-risk factors (lymphovascular space invasion (LVSI), depth of stromal invasion (DSI) and tumor size), adjuvant therapy is necessary when combinations of three factors meet the Sedlis criteria as follows: (a) positive LVSI, deep 1/3 DSI; (b) positive LVSI, middle 1/3 DSI, tumor size ≥ 2 cm; (c) positive LVSI, superficial 1/3 DSI, tumor size ≥ 5 cm; or (d) negative LVSI, middle or deep 1/3 DSI, tumor size ≥ 4 cm. Although the Sedlis criteria is widely used in the prognostic assessment of cervical cancer and the guidance of postoperative adjuvant treatment, its sensitivity in predicting recurrence is low, and it lacks consideration of histologic type.

To integrate the prognostic value of histologic type, based on a multicenter study, researchers redefined the intermediate-risk group using a ‘four-factor model’, in which the presence of any two factors (tumor size ≥ 3 cm, deep 1/3 DSI, LVSI, and adenocarcinoma or adenosquamous carcinoma histology) may be useful for predicting postoperative recurrence in cervical cancer patients. However, this model only adds the variable histologic type but does not take into account the unique tumor biological characteristics of adenocarcinoma, and its reliability with a C-index value of only 0.634 also needs further verification. Thus, it is urgent and necessary to explore a unique criterion to define the intermediate risk group to better predict postoperative recurrence and guide adjuvant therapy specific for AC patients.

Based on the above dilemma, a new Silva classification was proposed in 2013 based on the pattern of stromal invasion morphology [10]. Pattern A is characterized by well-demarcated glands with no destructive invasion or LVSI, pattern B represents localized destructive invasion, and pattern C demonstrates diffusely infiltrative glands. This new system was then validated in several subsequent studies [11–14] and showed better performance in predicting nodal metastasis and prognosis. Therefore, taking into consideration the Silva pattern for AC patients and establishing a Silva-based model based on the unique biological behavior of AC may help provide a novel prognostic estimation and postoperative adjuvant treatment decision-making system.

In the present study, we established various novel and unique Silva-based models to predict postoperative recurrence specific for the intermediate-risk grouping of patients with AC for the first time, including 12 four-factor models, 30 three-factor models and 16 two-factor models. Among them, the four-factor model, which included any three of four intermediate-risk factors (Silva C, ≥ 3 cm, DSI > 2/3, > mild LVSI), exhibited the best recurrence prediction performance. Notably, among the 4 combinations in the best model, when patients met the three factors of Silva C, ≥ 3 cm, and DSI > 2/3, the model exhibited the best discriminating ability for recurrence. These findings highlight the precise prognostic value of the Silva-based four-factor model we established, providing a unique prognostic estimation system and postoperative adjuvant treatment decision-making system specific for intermediate-risk AC patients.

## Materials and methods

### Study population

We identified 345 patients with a histology of usual AC from January 2006 to December 2017 at the Obstetrics and Gynecology Hospital of Fudan University as the study population. All patients received radical hysterectomy based on different stages in accordance with the NCCN guidelines at the time. Most patients underwent type C radical hysterectomy, except for four FIGO stage IA1(LVSI)-IA2 patients underwent type B radical hysterectomy. The exclusion criteria included patients with a history of prior malignancy, a preexisting history of chemotherapy or radiotherapy for other conditions, combination with other histologic types and death due to surgical complications.

### Data collection

For the eligible patients, patient demographics, laboratory test results, therapy data, tumor characteristics and survival outcomes were retrospectively collected from the Obstetrics and Gynecology Hospital of Fudan University. All medical records were reviewed simultaneously by three experts and independently checked by two experts to ensure accuracy.

The demographic variables included age and comorbidity (hypertension/diabetes). The laboratory test results included HPV infection status. The therapy data included surgical approach, operative time, blood loss, transfusion, history of loop electrosurgical excision procedure (LEEP) and adjuvant treatment. The tumor characteristics included International Federation of Gynecology and Obstetrics (FIGO) (2009) stage, tumor size, DSI, LVSI, surgical margin, parametrial involvement, and LN status. LN metastasis was classified as no metastasis, pelvic LNs, common iliac LNs and para-aortic LNs. If metastases were observed in two or more locations, then the furthest LN station was marked. For example, patients with positive para-aortic LNs and pelvic LN metastases were classified as having positive para-aortic LNs.

The primary outcomes were 3-year recurrence-free survival (RFS) and 3-year OS. RFS was defined as the interval from the initial cervical cancer diagnosis to the first finding of any recurrence or the last follow-up. OS was defined as the interval from the initial diagnosis to cervical cancer-related death or the last follow-up. Patients who failed to reach the survival events at the last follow-up were censored. Local recurrences were defined by pathologic proof of cancer in the vagina/cervix, which was confined to the pelvis, or an imaging study showing the regrowth of the tumor or an enlargement of any pelvic LN. Distant recurrences were defined as any recurrence outside of the pelvis, including peritoneal spread or the involvement of supraclavicular LNs, the lung, the liver, the bone, the brain, etc. based on pathologic, cytologic or radiologic evidence. The definition of local or distant recurrence was determined according to the lesions detected at the time of the first relapse after a complete workup.

### Histopathologic review

Surgical specimen slides from 345 AC patients with at least a 3-year follow-up were collected. A consensus diagnosis was reached according to the Silva classification based on the stromal invasion pattern, with at least 3 pathologists reviewing slides with a multi-head microscope for each case.

Pattern A tumors have a nondestructive pattern of stromal invasion without LVSI, and the glands are usually round, with complete outer contours, and distributed in groups in a lobular distribution. Pattern B tumors have early, small foci of destructive stromal invasion typically arising from Pattern A-type glands and sometimes show LVSI. The outline of the gland is generally damaged and infiltrated, with incomplete borders. There may be individual cancer cell clusters that dissociate in the gland, and tumor thrombi can be seen in the vasculature. Pattern C tumors have diffused destructive invasion and frequent LVSI. Intercellular junctions and cell polarization are often missing, and tumor cell invasion ability is enhanced.

The criteria for determining the grade of LVSI were as follows: none (no LVSI), mild (a single focus of LVSI was recognized around a tumor), and substantial (diffuse or multifocal LVSI were recognized around the tumor). We have added this information in method section.

### Clinical information

According to NCCN guidelines, preoperative workup for patients with suspicious symptoms includes history, physical examination, cervical cytologic screening, routine blood tests (including platelets), liver and renal function tests, ECG and imaging examinations. Radiologic imaging includes chest X-ray, pelvic CT/MRI, or combined PET-CT as indicated [15, 16]. Cone biopsy is used if the cervical biopsy is inadequate to define invasiveness or if accurate assessment of microinvasive disease is required. When patients were older than 60, echocardiography, pulmonary function tests and urodynamic tests were also needed.

All patients underwent modified radical hysterectomy or radical hysterectomy with bilateral pelvic lymphadenectomy with or without para-aortic lymphadenectomy according to NCCN guidelines. The patients underwent adjuvant treatment after radical hysterectomy when they met one of the following two criteria: (a) patients who presented any one of several high-risk factors (positive surgical margin, parametrial involvement, and LN metastasis) and (b) the Sedlis criteria were satisfied for intermediate-risk factors (tumor size, LVSI, and DSI). After hospital discharge, the patients received regular follow-up in accordance with the NCCN guidelines [9]. The median follow-up time was 102 (36–168) months.

### Statistical analysis

Continuous variables are reported as medians with interquartile ranges (IQRs) or means with standard deviations (SDs). Categorical variables are reported as numbers and proportions. We used Student’s t-test to compare continuous variables and Fisher’s exact test or the χ^2^ test to compare categorical variables. The collinearity of all variables was evaluated using correlation matrices, and no significant interaction was identified. The Kaplan–Meier method with the log-rank test was used to compare survival outcomes. The associations of variables with RFS and OS were evaluated using Cox proportional hazards regression models. Hazard ratios (HRs) are presented with 95% confidence intervals (CIs).

The statistical software used for analyses included SPSS (version 21.0; SPSS Inc., Chicago, IL, USA) and Python 3.7 (https://www.python.org/). All tests were two-sided, and *p* < 0.05 was considered statistically significant.

### Ethics

This retrospective study was granted ethical approval by the Institutional Ethics Committee of Fudan University Obstetrics and Gynecology Hospital (2020–22), informed consent was obtained from the participants. All methods were carried out in accordance with relevant guidelines and regulations.

## Results

### Baseline characteristics of 345 patients with AC

The characteristics of the 345 AC patients are presented in Additional file 1: Table S1. The median age was 46.4 years, and most patients were in stage I (89.6%). Of the 345 patients, there were 96 (27.8%) with pattern A, 90 (26.1%) with pattern B and 159 (46.1%) with pattern C. The median follow-up period was 102 (36–168) months, during which 27 (7.8%) patients died and 32 (9.3%) patients experienced recurrence. In terms of the recurrence site, there were 16 patients with initial recurrence in the local region and 16 patients with recurrence in the distant region.

### Prognostic value of the Silva classification in AC patients

The clinicopathological factors of the 345 patients were compared according to different Silva patterns (Table 1). There was no positive parametrial involvement, LVSI or perineural invasion (PNI) in patients with pattern A. Compared to patients with pattern A, patients with pattern B or C had higher FIGO stages (*p* < 0.001), larger tumor sizes (*p* < 0.001), and deeper stromal invasion (*p* < 0.001) and showed a higher frequency of positive LNs (*p* < 0.001), positive surgical margins (*p* = 0.043), positive parametrial involvement (*p* < 0.001), positive LVSI (*p* < 0.001) and positive PNI (*p* = 0.002). In addition, patients with pattern B or C were more likely to receive postoperative adjuvant therapy (*p* < 0.001) and undergo laparotomy (*p* = 0.033) than those with pattern A.

**Table 1:** Comparison of clinic-pathological factors between different Silva patterns

Characteristics	Pattern A (N = 96)	Pattern B (N = 90)	Pattern C (N = 159)	*p* value
Age			0.053	
Mean ± SD	44.8 ± 9.9	45.7 ± 8.9	47.8 ± 10.6	
FIGO stage (%)				< 0.001
Stage 1	93 (96.9)	86 (95.6)	130 (81.8)	
Stage 2	3 (3.1)	4 (4.4)	29 (18.2)	
Comorbidity (%)			0.904	
No	85 (88.5)	78 (86.7)	138 (86.8)	
Yes	11 (11.5)	12 (13.3)	21 (13.2)	
Adjuvant treatment (%)			< 0.001	
No	69 (71.9)	42 (46.7)	32 (65.9)	
Yes	27 (28.1)	48 (53.3)	127 (79.9)	
HPV infect (%)				0.004
No	1 (1)	2 (2.2)	9 (5.7)	
Yes	29 (30.2)	41 (45.6)	39 (24.5)	
Unknown	66 (68.8)	47 (52.2)	111 (69.8)	
LEEP (%)				0.403
No	91 (94.8)	85 (94.4)	155 (97.5)	
Yes	5 (5.2)	5 (5.6)	4 (2.5)	
Surgical approach (%)			0.033	
Laparoscopy	88 (91.7)	86 (95.6)	136 (85.5)	
Laparotomy	8 (8.3)	4 (4.4)	23 (14.5)	
Surgical duration, min (%)			0.23	
≤ 200	50 (52.1)	56 (62.2)	82 (51.6)	
> 200	46 (47.9)	34 (37.8)	77 (48.4)	
Blood loss, ml (%)			0.943	
≤ 200	51 (53.1)	49 (54.4)	83 (52.2)	
> 200	45 (46.9)	41 (45.6)	76 (47.8)	
Transfusion (%)				0.186
No	93 (96.9)	82 (91.1)	145 (91.2)	
Yes	3 (3.1)	8 (8.9)	14 (8.8)	
LN metastasis (%)			< 0.001	
No	94 (97.9)	85 (94.4)	109 (68.6)	
Yes	2 (2.1)	5 (5.6)	50 (31.4)	
Metastasis site (%)			< 0.001	
No	94 (97.9)	85 (94.4)	109 (68.6)	
Pelvic LN	2 (2.1)	5 (5.6)	32 (20.1)	
Common iliac LN	0 (0)	0 (0)	15 (9.4)	
Para-aortic LN	0 (0)	0 (0)	3 (1.9)	
Surgical margin (%)			0.043	
No	94 (97.9)	89 (98.9)	148 (93.1)	
Yes	2 (2.1)	1 (1.1)	11 (6.9)	
Parametrial invasion (%)			< 0.001	
No	96 (100)	89 (98.9)	140 (88.1)	
Yes	0 (0)	1 (1.1)	19 (11.9)	
Tumor size, cm (%)			< 0.001	
≤ 2	72 (75)	50 (55.6)	32 (20.1)	
(2,4)	23 (24)	33 (36.7)	90 (56.6)	
> 4	1 (1)	7 (7.8)	37 (23.3)	
LVSI (%)				< 0.001
No	96 (100)	67 (74.4)	63 (39.6)	
Mild	0 (0)	22 (24.4)	56 (35.2)	
Substantial	0 (0)	1 (1.1)	40 (25.2)	
DSI (%)				< 0.001
No	28 (29.2)	10 (11.1)	12 (7.5)	
< 2/3	61 (63.5)	55 (61.1)	27 (17)	
≥ 2/3	7 (7.3)	25 (27.8)	120 (75.5)	
PNI (%)				0.002
No	96 (100)	87 (96.7)	143 (89.9)	
Yes	0 (0)	3 (3.3)	16 (10.1)	

**Fig. 1:**
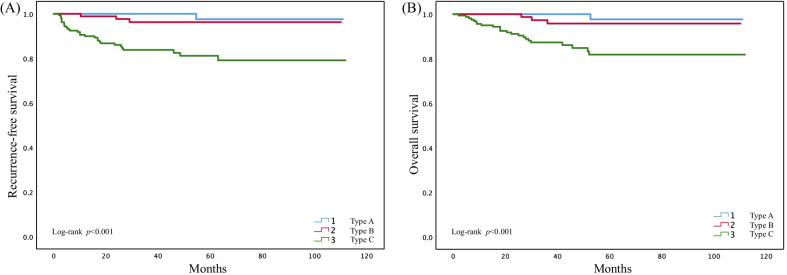
Survival outcome comparison according to different Silva patterns

We then compared the survival outcome according to different Silva patterns. Only 1 (1%) patient with pattern A experienced recurrence (a 70 years old patient, who underwent vagina recurrence after 52 months from surgery, and died 2 months later), while 3 (3.3%) with pattern B and 28 (17.6%) with pattern C experienced recurrence. In terms of death, 1 (1%) pattern A patient, 3 (3.3%) pattern B patients and 23 (14.5%) pattern C patients died during the follow-up period. The results of Kaplan–Meier survival analysis showed that patients with pattern C had the worst prognosis. The 3-year RFS rates for patterns A, B and C were 100%, 96.2% and 83.1%, respectively (*p* < 0.001), while the 3-year overall survival rates for patterns A, B and C were 100%, 95.8% and 86.7% (*p* < 0.001) (Fig. 1). The univariate Cox analysis also showed the same findings (Table 1). Compared to pattern A, pattern B (RFS: HR, 3.282 [0.341, 31.556], *p* < 0.001; OS: HR, 3.279 [0.341, 31.531], *p* < 0.001) or pattern C (HR, 18.936 [2.576, 139.2], *p* < 0.001; OS: HR, 15.472[2.089,114.607], *p* < 0.001) had significant worse prognosis. In addition, compared to pattern A + B, pattern C also showed worse RFS (HR, 9.06 [3.177, 25.836], *p* < 0.001) and OS (HR, 7.406 [2.56, 21.421], *p* < 0.001). Taken together, these results indicated that as the Silva grade increased, the patient's prognosis worsened”.

### Significant intermediate-risk factors in AC patients

In the cohort of 345 AC patients, univariate Cox analysis showed that in addition to Silva pattern (RFS, *p* < 0.001; OS, *p* = 0.002), the following 13 factors were significantly associated with RFS and OS (Table 2): age (RFS, *p* = 0.009; OS, *p* = 0.047), FIGO stage (RFS, *p* < 0.001; OS, *p* = 0.003), adjuvant therapy (RFS, *p* = 0.012; OS, *p* = 0.026), surgical approach (RFS, *p* < 0.019; OS, *p* = 0.008), transfusion (RFS, *p* = 0.015; OS, *p* = 0.005), LN metastasis (RFS, *p* < 0.001; OS, *p* < 0.001), LN metastasis site (RFS, *p* < 0.001; OS, *p* < 0.001), surgical margin (RFS, *p* = 0.001; OS, *p* < 0.001), parametrial involvement (RFS, *p* < 0.001; OS, *p* < 0.001), tumor size (RFS, *p* = 0.001; OS, *p* = 0.002), LVSI (RFS, *p* < 0.001; OS, *p* < 0.001), DSI (RFS, *p* < 0.001; OS, *p* < 0.001) and PNI (RFS, *p* = 0.005; OS, *p* = 0.001). Among these 14 factors, LN, surgical margin and parametrial involvement were identified as high-risk factors according to NCCN guidelines. Thus, we further excluded patients who had any one of the above three high-risk factors and focused on the remaining 254 patients as the intermediate-risk group in the following analysis.

**Table 2:** Univariate Cox analysis in 345 AC patients

Characteristics	Univariate Cox analysis (RFS)		Univariate Cox analysis (OS)	
	HR (95%CI)	*p* value	HR (95%CI)	*p* value
Age	0.009		0.047	
Mean ± SD	1.044 [1.011, 1.079]		2.152 [1.011, 4.58]	
FIGO stage (%)	< 0.001		0.003	
Stage 1	1		1	
Stage 2	3.98 [1.839, 8.611]		3.754 [1.584, 8.899]	
Silva (%)		< 0.001		0.002
Type A	1		1	
Type B	3.282 [0.341, 31.556]		3.279 [0.341, 31.531]	
Type C	18.936 [2.576, 139.2]		15.472 [2.089, 114.607]	
Comorbidity (%)	0.289		0.373	
No	1		1	
Yes	1.616 [0.665, 3.928]		1.555 [0.589, 4.108]	
Adjuvant treatment (%)	0.012		0.026	
No	1		1	
Yes	3.11 [1.28, 7.557]		3.017 [1.142, 7.967]	
LEEP (%)	0.703		0.443	
No	1		1	
Yes	0.679 [0.093, 4.974]		0.046 [0, 119.3]	
Surgical approach (%)	0.019		0.008	
Laparoscopy	1		1	
Laparotomy	2.623 [1.169, 5.89]		3.097 [1.344, 7.134]	
Surgical duration, min (%)	0.914		0.226	
≤ 200	1		1	
> 200	0.962 [0.478, 1.936]		0.616 [0.281, 1.35]	
Blood loss, ml (%)	0.975		0.996	
≤ 200	1		1	
> 200	0.989 [0.49, 1.995]		0.998 [0.465, 2.14]	
Transfusion (%)	0.015		0.005	
No	1		3.66 [1.475, 9.084]	
Yes	3.024 [1.241, 7.37]			
LN metastasis (%)	< 0.001		< 0.001	
No	1		1	
Yes	5.94 [2.969, 11.885]		8.349 [3.873, 17.997]	
LN metastasis site (%)	< 0.001		< 0.001	
No	1		1	
Pelvic	2.956 [1.156, 7.557]		4.254 [1.573, 11.506]	
Common iliac	15.596 [6.873, 35.388]		20.178 [8.35, 48.762]	
Para-aortic	12.111 [1.587, 92.455]		17.452 [2.178, 139.82]	
Surgical margin (%)	0.001		< 0.001	
No	1		1	
Yes	5.307 [2.041, 13.798]		7.697 [3.105, 19.078]	
Parametrial invasion (%)	< 0.001		< 0.001	
No	1		1	
Yes	5.516 [2.383, 12.768]		6.493 [2.743, 15.369]	
Tumor size, cm (%)	0.001		0.002	
≤ 2	2.198 [0.895, 5.396]		1	
(2,4)	5.834 [2.217, 15.354]		1.556 [0.603, 4.016]	
> 4			5.355 [1.991, 14.406]	
LVSI (%)		< 0.001		< 0.001
No	1		14.675 [4.176, 51.57]	
Mild	13.207 [4.411, 39.544]		25.267 [7.034, 90.757]	
Substantial	21.02[6.768,65.285]			
DSI (%)		< 0.001		< 0.001
< 2/3	1		1	
≥ 2/3	20.369 [4.868, 85.236]		7.463 [2.581, 21.581]	
PNI (%)		0.005		0.001
No	1		1	
Yes	3.955 [1.519, 10.293]		4.898 [1.848, 12.981]	

We then divided the remaining risk factors based on different cutoff values. For example, age was categorized as 40, 50 or 60 years old. Silva pattern was categorized as Silva B + C or Silva C. Tumor size was categorized as 2, 2.5, 3, 3.5, 4, 4.5 or 5 cm. DSI was categorized to > 1/3 DSI or > 2/3 DSI. LVSI was categorized as mild LVSI or substantial LVSI (Additional file 1: Table S2). Univariate Cox analysis showed that 5 variables had a *p*-value of less than 0.05 regarding to RFS: Silva C, ≥ 3 cm, ≥ 3.5 cm, DSI > 2/3 and > mild LVSI. In addition, considering the important prognostic value of Silva pattern, we also added Silva B + C, which had a critical *p*-value of 0.057, as a potential intermediate-risk variable. Thus, we focused on these 6 variables (Silva C, Silva B + C, ≥ 3 cm, ≥ 3.5 cm, DSI > 2/3 and > mild LVSI) (Table 3), which are also considered as 4 intermediate-risk factors (Silva pattern, diameter, DSI and LVSI), for the following study.

**Table 3:** Significant intermediate risk factors in 254 intermediate-risk AC patients

Intermediate risk factors	Univariate Cox Analysis		Multivariate Cox Analysis	
	HR, 95%CI	*p* value	HR, 95%CI	*p* value
Silva B + C	7.281 [0.946, 56.036]	0.057	1.194 [0.101, 14.093]	0.888
Silva C	5.455 [1.677, 17.746]	0.005	1.501 [0.411, 5.486]	0.539
≥ 3 cm	4.252 [1.391, 13]	0.011	2.141 [0.672, 6.823]	0.198
≥ 3.5 cm	3.212 [1.079, 9.562]	0.036	0.685 [0.137, 3.435]	0.646
DSI > 2/3	11.453 [2.538, 51.68]	0.002	7.696 [1.598, 37.051]	0.011
> mild LVSI	6.24 [2.018, 19.292]	0.001	3.185 [0.98, 10.356]	0.054

### Establishment of a novel Silva-based model specific for intermediate-risk AC patients

**Fig. 2:**
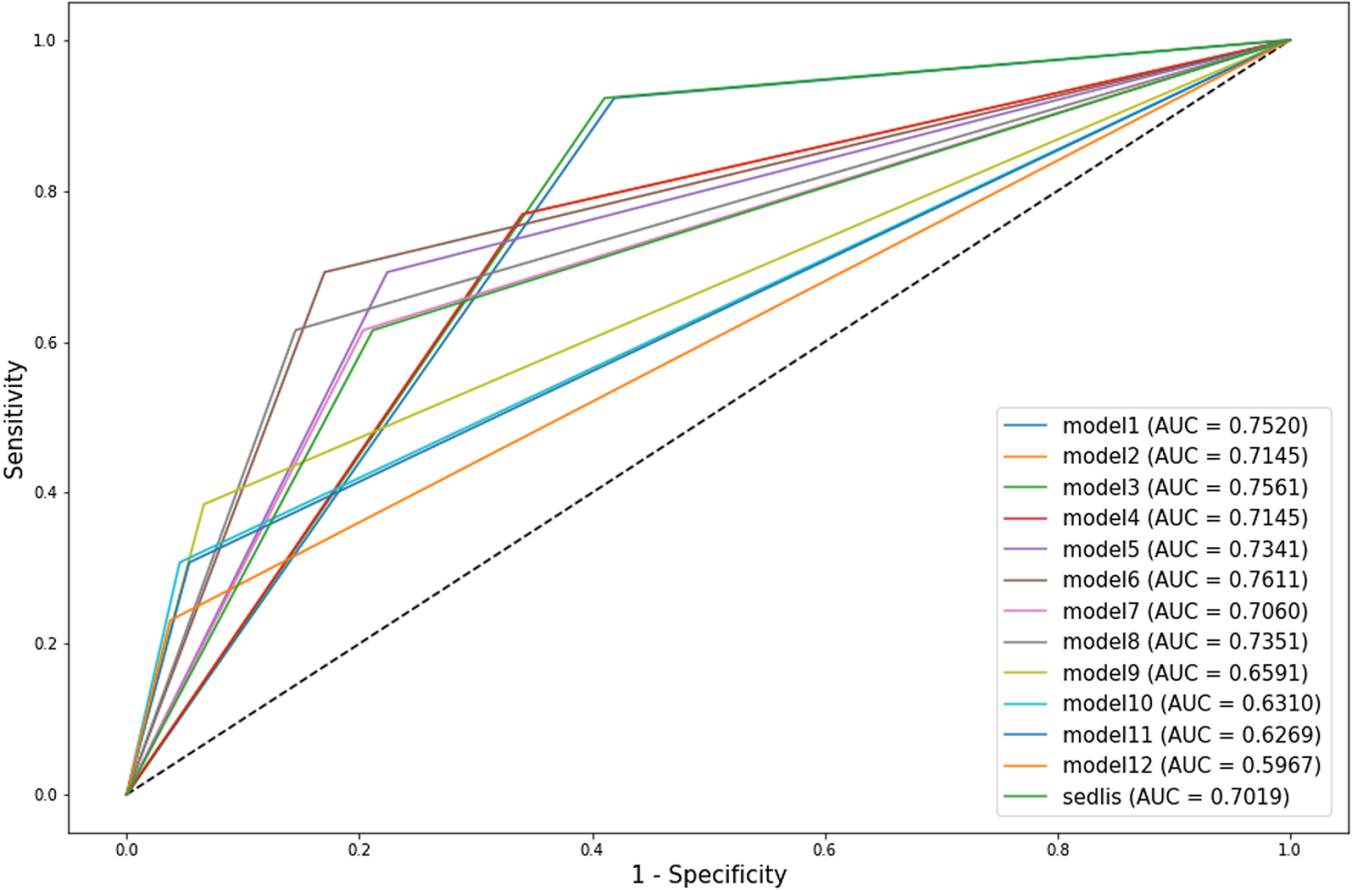
Four-factor model performance comparison

To explore a unique Silva-based model specific for intermediate-risk AC patients to better guide postoperative adjuvant therapy, we established various models using different combinations of the above 4 intermediate-risk factors (also considered the above 6 variables: Silva C, Silva B + C, ≥ 3 cm, ≥ 3.5 cm, DSI > 2/3 and > mild LVSI), which exhibited a significant association with RFS, and compared the model performance with that of the traditional Sedlis criteria. First, we established 12 models via various combinations of the four intermediate-risk factors, including the above 6 variables (Table 4). Interestingly, most four-factor models had superior performance to the traditional Sedlis criteria. Of note, among the 12 four-factor models (Fig. 2), model 6 (any 3 of the 4 factors: Silva C, ≥ 3 cm, DSI > 2/3, and > mild LVSI) showed the best recurrence prediction performance, the highest chi-square score (24.262), and the highest area under the curve (AUC) value (0.761), and was significant in univariate Cox analysis (HR, 10.792 [3.31, 35.19], *p* < 0.001).

**Table 4:** Four-factor model performance comparison

	Univariate Cox Analysis			Log-rank test		C index	95%CI
Models	*p* value	HR	95% CI	Chi-Square	*p* value		
Sedlis Criteria	0.002	6.137	2–18.833	13.083	0.000	0.702	0.597–0.78
Four-factor Model: Tumor size, DSI, LVSI, Silva							
*Any 2 of 4 factors*							
Silva B + C, ≥ 3 cm, DSI > 2/3, > mild LVSI	0.008	16.028	2.083–123.36	12.964	0.000	0.752	0.676–0.804
Silva C, ≥ 3 cm, DSI > 2/3, > mild LVSI	0.005	6.507	1.787–23.694	10.684	0.001	0.715	0.606–0.798
Silva B + C, ≥ 3.5 cm, DSI > 2/3, > mild LVSI	0.007	16.676	2.166–128.37	13.55	0.000	0.756	0.676–0.809
Silva C, ≥ 3.5 cm, DSI > 2/3, > mild LVSI	0.005	6.507	1.787–23.694	10.684	0.001	0.715	0.606–0.798
*Any 3 of 4 factors*							
Silva B + C, ≥ 3 cm, DSI > 2/3, > mild LVSI	0.001	7.714	2.37–25.105	16.07	0.000	0.734	0.640–0.837
Silva C, ≥ 3 cm, DSI > 2/3, > mild LVSI	< 0.001	10.792	3.31–35.19	24.262	0.000	0.761	0.670–0.862
Silva B + C, ≥ 3.5 cm, DSI > 2/3, > mild LVSI	0.001	6.321	2.059–19.402	13.621	0.000	0.706	0.595–0.799
Silva C, ≥ 3.5 cm, DSI > 2/3, > mild LVSI	< 0.001	9.445	3.065–29.101	22.696	0.000	0.735	0.626–0.830
*All 4 factors*							
Silva B + C, ≥ 3 cm, DSI > 2/3, > mild LVSI	< 0.001	8.526	2.768–26.263	20.084	0.000	0.659	0.554–0.804
Silva C, ≥ 3 cm, DSI > 2/3, > mild LVSI	< 0.001	8.143	2.502–26.505	17.267	0.000	0.631	0.525–0.764
Silva B + C, ≥ 3.5 cm, DSI > 2/3, > mild LVSI	0.001	7.251	2.223–23.645	14.792	0.000	0.627	0.523–0.770
Silva C, ≥ 3.5 cm, DSI > 2/3, > mild LVSI	0.005	6.46	1.776–23.493	10.632	0.001	0.597	0.519–0.696

Moreover, given the best performance of model 6 (any 3 of the 4 factors: Silva C, ≥ 3 cm, DSI > 2/3, and > mild LVSI), we wanted to further determine which combination in model 6 showed the best predictive ability for recurrence. To accomplish this, we compared the 4 combinations in model 6 based on any 3 of the 4 factors. The results showed that when patients met the three factors of Silva C, ≥ 3 cm, and DSI > 2/3, the model exhibited the best discriminating ability for recurrence (Additional file 1: Table S3, Additional file 2: Fig. S1).

We also established 30 three-factor models (Additional file 1: Table S4, Additional file 3: Fig. S2) and 16 two-factor models (Additional file 1: Table S5, Additional file 4: Fig. S3) by replacing one or two of the three intermediate risk factors (tumor size, LVSI, and DSI) with Silva patern. The results showed that, despite being inferior to the four-factor models, 8 three-factor and 5 two-factor models were better than the Sedlis criteria. This again demonstrated the important recurrence prediction value of Silva pattern.

## Discussion

In this study, (1) we confirmed the prognostic value of Silva pattern using a Chinese cohort of 345 AC patients. (2) We established Silva pattern-based models with potential recurrence prediction value in 254 intermediate-risk AC patients, including 12 four-factor models, 30 three-factor models and 16 two-factor models. (3) Notably, compared with the conventional Sedlis criteria, the four-factor model, which includes any three of four intermediate-risk factors (Silva C, ≥ 3 cm, DSI > 2/3, and > mild LVSI), exhibited better recurrence prediction performance and surpassed the other three-factor and two-factor models. (4) Specifically, in the best model (any three of the four factors: Silva C, ≥ 3 cm, DSI > 2/3, and > mild LVSI), we further constructed various combinations and found that the model exhibited the best discriminating ability for recurrence when patients met the three factors of Silva C, ≥ 3 cm, and DSI > 2/3. In conclusion, our findings highlight the prognostic value of the Silva pattern and provide a novel and unique Silva-based recurrence prediction model specific for AC patients with intermediate-risk factors, which may not only guide postoperative adjuvant treatment but also precisely provide shunting guidance based on histology.

AC is a unique histologic type of cervical cancer with an increasing incidence in recent years. Compared to SCC, AC has a distinct epidemiology, higher LN metastasis rate and worse prognosis. However, the treatment modalities for the two histologic types are the same according to NCCN guidance, which has been controversial, especially for cervical cancer patients with intermediate-risk factors after radical hysterectomy. According to classic criteria [17–19], patients are recommended to receive adjuvant therapy if they have any two of the following three factors: 2 cm, LVSI, and mild DSI. However, the specificity for predicting recurrence and survival is low. Then, the Gynecologic Oncology Group (GOG) defined the Sedlis criteria [20], which includes various combinations of three factors (LVSI, DSI, and tumor size). Despite its extensive use in clinical practice, the Sedlis criteria exhibited low sensitivity in predicting recurrence and survival and does not take into account the histologic type. Thus, a new model that includes any two of four intermediate-risk factors (3 cm, LVSI, outer DSI, and adenocarcinoma) was developed in 2014. Nevertheless, this model has a relatively low C index (0.634) and only adds the variable adenocarcinoma but lacks the consideration of the unique tumor biological characteristics of each histologic type. Therefore, a unique standard needs to be established for intermediate-risk AC patients to better guide postoperative adjuvant therapy and improve AC patient prognostic outcomes.

The Silva system is a new pattern-based classification that is categorized as A, B or C based on stromal invasion morphology. This promising classification was shown to have a better prognostic value in predicting LN metastasis [10, 13, 21]. Consistent with previous findings, our results also demonstrated that Silva pattern has significant prognostic value. As the Silva grade increased, the 3-year RFS and OS worsened. Specifically, there was no positive parametrial involvement, no LVSI and no PNI in patients with pattern A, which is similar to the findings of other studies [10, 11, 21].

Considering the deficiencies of previous criteria and the promising prognostic value of Silva pattern for AC patients, in the present study, we considered the Silva pattern for AC patients and established new Silva-based models specific for the intermediate-risk group of AC patients. Among all models, the four-factor model, which defines the intermediate-risk group according to the presence of any three of four risk variables (Silva C, ≥ 3 cm, DSI > 2/3, and > mild LVSI), showed better recurrence discriminating ability than the conventional Sedlis criteria and surpassed the other three-factor and two-factor models in terms of performance. We speculated that the improvement in prediction performance may be due to the inclusion of Silva pattern, which can reflect the unique characteristics of AC and was ignored in previous studies. Of note, although ‘2 cm’ is associated with the recurrence rate [22, 23], our results demonstrated that ‘3 cm’ might be a better prognostic factor to predict recurrence in AC patients, which is similar to Ryu’s findings [24].

Another interesting finding was that in the multivariate Cox analysis regarding to RFS in 254 intermediate-risk AC patients, only ‘DSI > 2/3’ was statistically significant. In addition, in the four-factor model (any three of four intermediate-risk factors: Silva C, ≥ 3 cm, DSI > 2/3, and > mild LVSI), three combinations that included ‘DSI’ have better prediction performance than ‘Silva C, ≥ 3 cm and > mild LVSI’. We speculate that it may be due to the following reasons: (1) Unlike SCC, AC has its unique biological behavior and usually exhibited an endophytic growth pattern with a barrel-shape cervix. Thus, considering its growth pattern, depth of invasion might outweigh tumor size, LVSI and Silva pattern in affecting patients’ prognosis. (2) Measuring depth of invasion in cervical adenocarcinoma poses challenges in clinical practice. Different from SCC which has a defined basement membrane, beyond which invasion is obvious, endocervical glands lack such landmark. The endocervical mucosa is a series of invaginations along the canal throughout the cervical wall, thus, making the determination of exact invasion origin rather difficult. Noteworthy, previous studies demonstrated that different measurements by at least one reviewer was sufficient to change the FIGO stage [25], potentially affecting treatment modalities, and thereby further affecting prognosis.

There are several limitations in this study. First, considering the retrospective nature of this study, future prospective studies are still warranted to further verify the reliability of the model. Second, our models were developed and validated based on Chinese patients, and the generalizability needs further validation with non-Chinese patient data. Third, as the follow-up time was relatively short (< 5 years), caution should be taken in applying this model to estimate long-term prognostic outcomes.

In conclusion, our study confirmed the prognostic value of Silva pattern in a cohort of 345 AC patients and, for the first time, constructed various Silva-based models to predict postoperative recurrence specific for the intermediate-risk group of patients with AC, including 12 four-factor models, 30 three-factor models and 16 two-factor models. Of note, the four-factor model, which includes any three of four intermediate-risk factors (Silva C, ≥ 3 cm, DSI > 2/3, and > mild LVSI), exhibited the best prediction ability and surpassed the conventional Sedlis criteria and the other three-factor and two-factor models. Among the 4 combinations in the best model, when patients met the three factors of Silva C, ≥ 3 cm, and DSI > 2/3, the model exhibited the best discriminating ability for recurrence. Our findings highlighted the prognostic value of Silva pattern, provided a special prognostic estimation system and offered guidance on postoperative treatment for intermediate-risk AC patients.

## Supplementary Information

**Additional file 1: Table S1.** Baseline characteristics of 345 patients with cervical adenocarcinoma. **Table S2.** The identification of cut-off value for intermediate risk factors in AC patients. **Table S3.** Performance comparison in best model. **Table S4.** Three-factor model performance comparison. **Table S5.** Three-factor model performance comparison

**Additional file 2: Fig. S1.** Four combinations in the best model 6

**Additional file 3. Fig. S2.** Three-factor model performance comparison

**Additional file 4: Fig. S3.** Two-factor model performance comparison

## Data Availability

The datasets used during the current study are available from the corresponding author on reasonable request.

## Abbreviations

HPV: Human papillomavirus
AC: Adenocarcinoma
SCC: Squamous cell carcinoma
NCCN: National comprehensive cancer network
LVSI: Lymphovascular space invasion
DSI: Depth of stromal invasion
PNI: Perineural invasion
LN: Lymph node
LEEP: Loop electrosurgical excision procedure
FIGO: Federation of gynecology and obstetrics
OS: Overall survival
RFS: Recurrence free survival
IQRs: Interquartile ranges
SDs: Standard deviations
HRs: Hazard ratios
CIs: Confidence intervals
AUC: Area under the curve (AUC)
GOG: Gynecologic oncology group
