# Supplementary material for: A novel Silva pattern-based model for precisely predicting recurrence in intermediate-risk cervical adenocarcinoma patients
Source: BMC Womens Health. 2022 Sep 16;22:377. doi: 10.1186/s12905-022-01971-z (PMC9482255; doi:10.1186/s12905-022-01971-z)
Supplement: Supplementary file 1 — Additional file 1: Table S1. Baseline characteristics of 345 patients with cervical adenocarcinoma. Table S2. The identification of cut-off value for intermediate risk factors in AC patients. Table S3. Performance comparison in best model. Table S4. Three-factor model performance comparison. Table S5. Three-factor model performance comparison [file 12905_2022_1971_MOESM1_ESM.docx]

**Supplementary appendix:**

**S1 Table.**

Baseline characteristics of 345 patients with cervical adenocarcinoma

**S2 Table.**

The identification of cut-off value for intermediate risk factors in AC patients

**S3 Table.**

Performance comparison in best model

**S4 Table.**

Three-factor model performance comparison

**S5 Table.**

Two-factor model performance comparison

**S1 Fig.**

Four combinations in the best model 6

**S2 Fig.**

Three-factor model performance comparison

**S3 Fig.**

Two-factor model performance comparison

**Appendix_supplementary tables:**

**Supplementary Table 1.**

Baseline characteristics of 345 patients with cervical adenocarcinoma

| Characteristics | Number (N=345) |
| --- | --- |
| Age |  |
| Mean ± SD | 46.4±10.1 |
| FIGO stage (%) |  |
| Stage 1 | 309 (89.6) |
| Stage 2 | 36 (10.4) |
| Silva (%) |  |
| Type A | 96 (27.8%) |
| Type B | 90 (26.1) |
| Type C | 159 (46.1) |
| Comorbidity (%) |  |
| No | 301 (87.2) |
| Yes | 44 (12.8) |
| Adjuvant treatment (%) |  |
| No | 143 (41.4) |
| Yes | 202 (58.6) |
| HPV infect (%) |  |
| No | 12 (3.5) |
| Yes | 109 (31.6) |
| Unknown | 224 (64.9) |
| LEEP (%) |  |
| No | 331 (95.9) |
| Yes | 14 (4.1) |
| Surgical approach (%) |  |
| Laparoscopy | 310 (89.9) |
| Laparotomy | 35 (10.1) |
| Surgical duration, min (%) |  |
| ≤ 200 | 188 (54.5) |
| >200 | 157 (45.5) |
| Blood loss, ml (%) |  |
| ≤ 200 | 183 (53) |
| >200 | 162 (47) |
| Transfusion (%) |  |
| No | 320 (92.8) |
| Yes | 25 (7.2) |
| LN metastasis (%) |  |
| No | 288 (83.5) |
| Yes | 57 (16.5) |
| Metastasis site (%) |  |
| No | 288 (83.5) |
| Pelvic LN | 39 (11.3) |
| Common iliac LN | 15 (4.3) |
| Para-aortic LN | 3 (0.9) |
| Surgical margin (%) |  |
| No | 331 (95.9) |
| Yes | 14 (4.1) |
| Parametrial invasion (%) |  |
| No | 325 (94.2) |
| Yes | 20 (5.8) |
| Tumor size, cm (%) |  |
| ≤2 | 154 (44.6) |
| (2,4] | 146 (42.4) |
| >4 | 45 (13) |
| LVSI (%) |  |
| No | 226 (65.5) |
| Mild | 78 (22.6) |
| Substantial | 41 (11.9) |
| DSI (%) |  |
| No | 50 (14.5) |
| <2/3 | 143 (41.4) |
| ≥2/3 | 152 (44.1) |
| PNI (%) |  |
| No | 326 (94.5) |
| Yes | 19 (5.5) |

**Supplementary Table 2.**

The identification of cut-off value for intermediate risk factors in AC patients

| Characteristics | Number, % | Univariate Cox analysis (RFS) | |
| --- | --- | --- | --- |
|  | (N=254) | HR (95%CI) | *p* value |
| Age60 |  |  | 0.295 |
| <60 | 236 (92.9) | 1 |  |
| ≥60 | 18 (7.1) | 2.239[0.496,10.108] |  |
| Age50 |  |  | 0.055 |
| <50 | 178 (68.7) | 1 |  |
| ≥50 | 76 (29.3) | 2.907 [0.977,8.653] |  |
| Age40 |  |  | 0.316 |
| <40 | 68 (26.8) | 1 |  |
| ≥40 | 186 (73.2) | 2.162[0.479,9.762] |  |
| Adjuvant treatment |  |  | 0.436 |
| No | 129 (50.8) | 1 |  |
| Yes | 125 (49.2) | 1.56[0.51,4.77] |  |
| Surgical approach |  |  | 0.773 |
| Laparoscopy | 234 (92.1) | 1 |  |
| Laparotomy | 20 (7.9) | 0.739[0.095,5.76] |  |
| Transfusion |  |  | 0.16 |
| No | 240 (94.5) | 1 |  |
| Yes | 14 (5.5) | 2.95[0.652,13.357] |  |
| Silva B+C |  |  | 0.057 |
| No | 92 (35.5) | 1 |  |
| Yes | 162 (62.5) | 7.281 [0.946,56.036] |  |
| Silva C |  |  | 0.005 |
| No | 175 (67.6) | 1 |  |
| Yes | 79 (30.5) | 5.455 [1.677,17.746] |  |
| ≥ 2cm |  |  | 0.248 |
| No | 119 (45.9) | 1 |  |
| Yes | 135 (52.1) | 2.002 [0.616,6.502] |  |
| ≥ 2.5 cm |  |  | 0.062 |
| No | 146 (56.4) | 1 |  |
| Yes | 108 (41.7) | 3.069 [0.944,9.979] |  |
| ≥ 3 cm |  |  | 0.011 |
| No | 181 (69.9) | 1 |  |
| Yes | 73 (28.2) | 4.252 [1.391,13] |  |
| ≥ 3.5 cm |  |  | 0.036 |
| No | 198 (76.4) | 1 |  |
| Yes | 56 (21.6) | 3.212 [1.079，9.562] |  |
| ≥ 4 cm |  |  | 0.069 |
| No | 220 (84.9) | 1 |  |
| Yes | 34 (13.1) | 2.986 [0.919,9.702] |  |
| ≥ 4.5 cm |  |  | 0.206 |
| No | 236 (91.1) | 1 |  |
| Yes | 18 (6.9) | 2.645 [0.585,11.956] |  |
| ≥ 5 cm |  |  | 0.6 |
| No | 242 (95.3) | 1 |  |
| Yes | 12 (4.7) | 4.271[0.943,19.347] |  |
| DSI>1/3 |  |  | 0.055 |
| No | 138 (53.3) | 1 |  |
| Yes | 116 (44.8) | 82.257 [0.911,74.25] |  |
| DSI>2/3 |  |  | <0.001 |
| No | 171 (66) | 1 |  |
| Yes | 83 (32) | 11.453 [2.538,51.68] |  |
| >mild LVSI |  |  | <0.001 |
| No | 195 (75.3) | 1 |  |
| Yes | 59 (22.8) | 6.24 [2.018,19.292] |  |
| >substantial LVSI |  |  | 0.552 |
| No | 242 (93.4) | 1 |  |
| Yes | 12 (4.6) | 1.86 [0.241,14.382] |  |
| PNI |  |  | 0.691 |
| No |  | 1 |  |
| Yes |  | 0.048[0,177] |  |

**Supplementary Table 3.**

Performance comparison in best model

|  | Univariate Cox Analysis | | | Log-rank test | | C index | 95%CI |
| --- | --- | --- | --- | --- | --- | --- | --- |
| Combinations | *p* value | HR | 95% CI | Chi-Square | *p* value |  |  |
| Any 3 of 4 factors: Silva C, ≥3 cm, DSI >2/3, >mild LVSI | |  |  |  |  |  |  |
| Silva C, ≥3 cm, DSI >2/3 | <0.001 | 10.925 | 3.668-32.543 | 29.031 | <0.001 | 0.726 | 0.609-0.841 |
| Silva C, ≥3 cm, >mild LVSI | 0.003 | 5.831 | 1.791-18.982 | 11.027 | 0.001 | 0.621 | 0.515-0.759 |
| Silva C, DSI >2/3, >mild LVSI | 0.002 | 6.115 | 1.994-18.752 | 13.068 | <0.001 | 0.649 | 0.537-0.760 |
| ≥3 cm, DSI >2/3, >mild LVSI | <0.001 | 8.526 | 2.768-26.263 | 20.084 | <0.001 | 0.659 | 0.554-0.804 |

**Supplementary Table 4.**

Three-factor model performance comparison

|  | Univariate Cox Analysis | | | Log-rank test | | C index | 95%CI |
| --- | --- | --- | --- | --- | --- | --- | --- |
| Models | *p* value | HR | 95% CI | Chi-Square | *p* value |  |  |
| Three-factor Model: Silva B+C, ≥3 cm, DSI >2/3 | |  |  |  |  |  |  |
| Any 1 of 3 factors | 0.165 | 35.012 | NC | 5.464 | 0.019 | 0.654 | 0.628-0.674 |
| Any 2 of 3 factors | 0.011 | 5.356 | 1.474-19.466 | 8.169 | 0.004 | 0.698 | 0.596-0.795 |
| All 3 factors | <0.001 | 10.447 | 3.414-31.969 | 26.187 | <0.001 | 0.746 | 0.625-0.873 |
| Three-factor Model: Silva C, ≥3 cm, DSI >2/3 | |  |  |  |  |  |  |
| Any 1 of 3 factors | 0.019 | 11.51 | 1.497-88.52 | 8.857 | 0.003 | 0.715 | 0.642-0.773 |
| Any 2 of 3 factors | 0.004 | 5.688 | 1.75-18.484 | 10.671 | 0.001 | 0.707 | 0.608-0.812 |
| All 3 factors | <0.001 | 10.925 | 3.668-32.543 | 29.031 | <0.001 | 0.726 | 0.606-0.841 |
| Three-factor Model: Silva B+C, ≥3.5 cm, DSI >2/3 | |  |  |  |  |  |  |
| Any 1 of 3 factors | 0.151 | 36.659 | NC | 5.974 | 0.015 | 0.664 | 0.642-0.686 |
| Any 2 of 3 factors | 0.008 | 5.81 | 1.598-21.119 | 9.178 | 0.002 | 0.706 | 0.603-0.798 |
| All 3 factors | <0.001 | 7.074 | 2.375-21.07 | 16.803 | <0.001 | 0.681 | 0.569-0.784 |
| Three-factor Model: Silva C, ≥3.5 cm, DSI >2/3 | |  |  |  |  |  |  |
| Any 1 of 3 factors | 0.014 | 12.893 | 1.676-99.159 | 10.126 | 0.001 | 0.729 | 0.654-0.786 |
| Any 2 of 3 factors | 0.003 | 6.073 | 1.868-19.739 | 11.702 | 0.001 | 0.713 | 0.616-0.820 |
| All 3 factors | <0.001 | 7.681 | 2.509-23.511 | 17.82 | <0.001 | 0.659 | 0.558-0.763 |
| Three-factor Model: Silva B+C, ≥3 cm, >mild LVSI | |  |  |  |  |  |  |
| Any 1 of 3 factors | 0.095 | 5.696 | 0.74-43.81 | 3.572 | 0.059 | 0.623 | 0.535-0.676 |
| Any 2 of 3 factors | 0.001 | 11.636 | 2.572-52.652 | 16.27 | <0.001 | 0.761 | 0.668-0.843 |
| All 3 factors | 0.003 | 5.505 | 1.79-16.928 | 11.193 | 0.001 | 0.640 | 0.531-0.790 |
| Three-factor Model: Silva C, ≥3 cm, >mild LVSI | |  |  |  |  |  |  |
| Any 1 of 3 factors | 0.018 | 6.151 | 1.363-27.763 | 7.29 | 0.007 | 0.689 | 0.592-0.771 |
| Any 2 of 3 factors | <0.001 | 11.377 | 3.107-41.654 | 21.266 | <0.001 | 0.766 | 0.663-0.851 |
| All 3 factors | 0.003 | 5.831 | 1.791-18.982 | 11.027 | 0.001 | 0.621 | 0.515-0.759 |
| Three-factor Model: Silva B+C, ≥3.5 cm, >mild LVSI | |  |  |  |  |  |  |
| Any 1 of 3 factors | 0.08 | 6.204 | 0.807-47.723 | 4.028 | 0.045 | 0.634 | 0.547-0.680 |
| Any 2 of 3 factors | 0.002 | 7.889 | 2.16-28.809 | 13.665 | <0.001 | 0.733 | 0.611-0.833 |
| All 3 factors | 0.008 | 5 | 1.53-16.335 | 8.754 | 0.003 | 0.612 | 0.507-0.756 |
| Three-factor Model: Silva C, ≥3.5 cm, >mild LVSI | |  |  |  |  |  |  |
| Any 1 of 3 factors | 0.012 | 6.902 | 1.529-31.159 | 8.525 | 0.004 | 0.703 | 0.611-0.783 |
| Any 2 of 3 factors | <0.001 | 8.506 | 2.593-27.901 | 17.796 | <0.001 | 0.736 | 0.611-0.820 |
| All 3 factors | 0.012 | 5.264 | 1.445-19.18 | 7.934 | 0.005 | 0.591 | 0.511-0.686 |
| Three-factor Model: Silva B+C, DSI >2/3, >mild LVSI | |  |  |  |  |  |  |
| Any 1 of 3 factors | 0.13 | 39.729 | NC | 6.881 | 0.009 | 0.678 | 0.652-0.699 |
| Any 2 of 3 factors | 0.004 | 9.363 | 2.072-42.309 | 12.574 | <0.001 | 0.743 | 0.660-0.827 |
| All 3 factors | 0.002 | 5.843 | 1.951-17.499 | 12.768 | <0.001 | 0.667 | 0.552-0.816 |
| Three-factor Model: Silva C, DSI >2/3, >mild LVSI | |  |  |  |  |  |  |
| Any 1 of 3 factors | 0.055 | 81.072 | NC | 15.839 | <0.001 | 0.782 | 0.755-0.809 |
| Any 2 of 3 factors | 0.001 | 8.59 | 2.356-31.32 | 15.303 | <0.001 | 0.746 | 0.634-0.830 |
| All 3 factors | 0.002 | 6.115 | 1.994-18.752 | 13.068 | <0.001 | 0.649 | 0.537-0.760 |

**Supplementary Table 5.**

Two-factor model performance comparison

|  | Univariate Cox Analysis | | | Log-rank test | | C index | 95%CI |
| --- | --- | --- | --- | --- | --- | --- | --- |
| Models | *p* value | HR | 95% CI | Chi-Square | *p* value |  |  |
| Two-factor Model: Silva B+C, ≥3 cm | |  |  |  |  |  |  |
| Any 1 of 2 factors | 0.095 | 5.696 | 0.74-43.81 | 3.572 | 0.059 | 0.623 | 0.535-0.676 |
| All 2 factors | 0.002 | 5.744 | 1.878-17.571 | 12.027 | 0.001 | 0.7 | 0.595-0.824 |
| Two-factor Model: Silva C, ≥3 cm |  |  |  |  |  |  |  |
| Any 1 of 2 factors | 0.024 | 4.4 | 1.211-15.992 | 6.06 | 0.014 | 0.671 | 0.575-0.760 |
| All 2 factors | <0.001 | 7.491 | 2.512-22.339 | 18.059 | <0.001 | 0.703 | 0.581-0.820 |
| Two-factor Model: Silva B+C, ≥3.5 cm |  |  |  |  |  |  |  |
| Any 1 of 2 factors | 0.08 | 6.24 | 0.807-47.723 | 4.028 | 0.045 | 0.634 | 0.547-0.680 |
| All 2 factors | 0.012 | 4.071 | 1.366-12.133 | 7.456 | 0.006 | 0.644 | 0.535-0.737 |
| Two-factor Model: Silva C, ≥3.5 cm |  |  |  |  |  |  |  |
| Any 1 of 2 factors | 0.014 | 5.022 | 1.382-18.251 | 7.428 | 0.006 | 0.688 | 0.592-0.773 |
| All 2 factors | 0.003 | 5.388 | 1.756-16.535 | 10.898 | 0.001 | 0.640 | 0.542-0.738 |
| Two-factor Model: Silva B+C, >mild LVSI |  |  |  |  |  |  |  |
| Any 1 of 2 factors | 0.057 | 7.281 | 0.946-56.036 | 4.994 | 0.025 | 0.650 | 0.561-0.698 |
| All 2 factors | 0.001 | 6.24 | 2.018-19.292 | 13.143 | <0.001 | 0.702 | 0.579-0.804 |
| Two-factor Model: Silva C, >mild LVSI |  |  |  |  |  |  |  |
| Any 1 of 2 factors | 0.003 | 9.665 | 2.134-43.766 | 12.978 | <0.001 | 0.738 | 0.647-0.819 |
| All 2 factors | 0.002 | 5.549 | 1.852-16.626 | 11.859 | 0.001 | 0.664 | 0.539-0.759 |
| Two-factor Model: Silva B+C, DSI >2/3, | |  |  |  |  |  |  |
| Any 1 of 2 factors | 0.13 | 39.729 | NC | 6.881 | 0.009 | 0.678 | 0.652-0.699 |
| All 2 factors | 0.002 | 8.031 | 2.209-29.191 | 14.185 | <0.001 | 0.746 | 0.641-0.833 |
| Two-factor Model: Silva C, DSI >2/3 |  |  |  |  |  |  |  |
| Any 1 of 2 factors | 0.006 | 17 | 2.21-130.758 | 13.892 | <0.001 | 0.762 | 0.686-0.822 |
| All 2 factors | 0.001 | 6.269 | 2.049-19.178 | 13.597 | <0.001 | 0.712 | 0.627-0.817 |
