# Supplementary figures and images for: A novel Silva pattern-based model for precisely predicting recurrence in intermediate-risk cervical adenocarcinoma patients
Source: BMC Womens Health. 2022 Sep 16;22:377. doi: 10.1186/s12905-022-01971-z (PMC9482255; doi:10.1186/s12905-022-01971-z)

**Supplementary Fig.1**

Four combinations in the best model 6

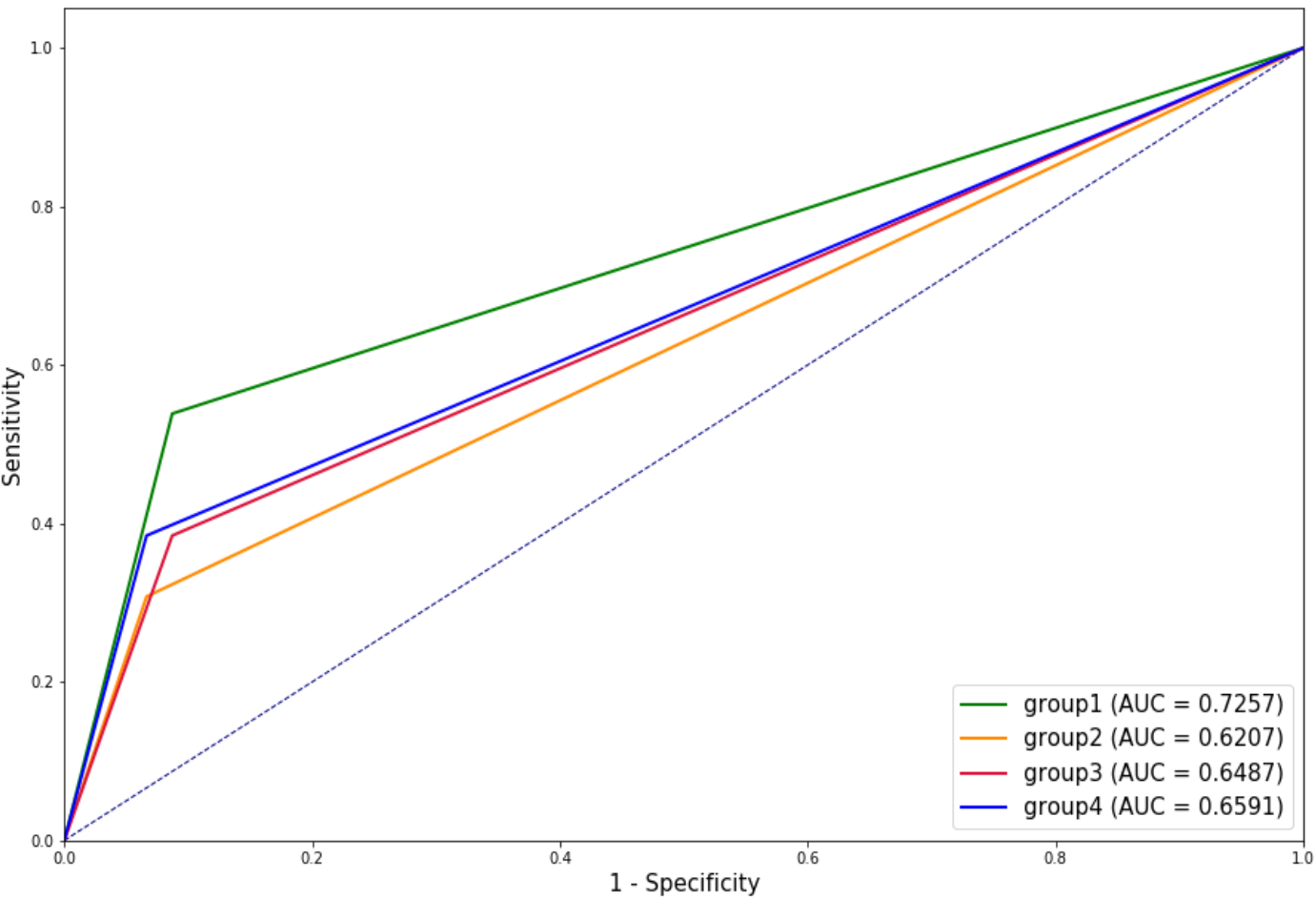

Supplement: Supplementary file 2 — Additional file 2: Fig. S1. Four combinations in the best model 6 [file 12905_2022_1971_MOESM2_ESM.pdf]

**Supplementary Fig 2**  
Three-factor model performance comparison

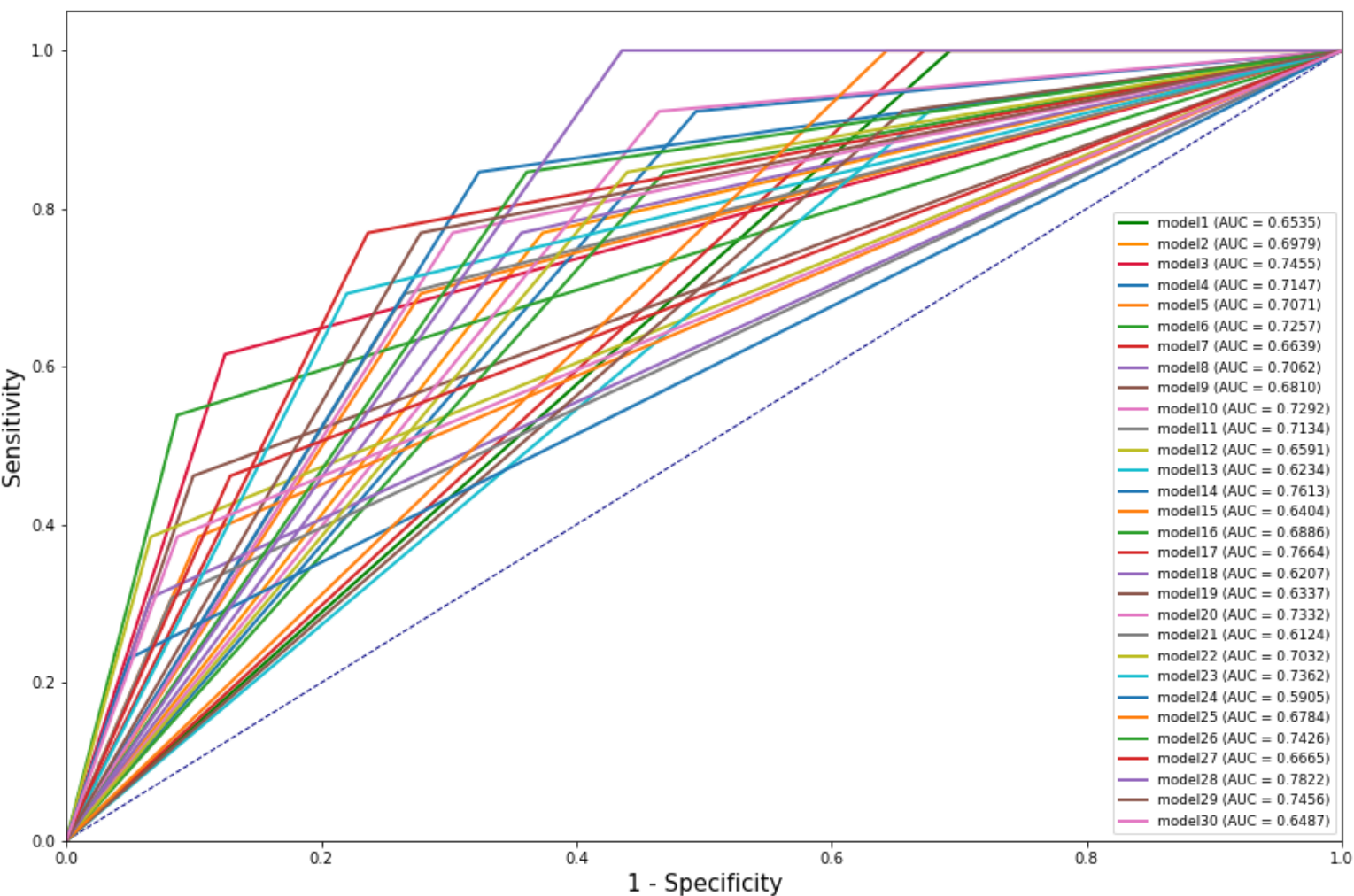

Supplement: Supplementary file 3 — Additional file 3. Fig. S2. Three-factor model performance comparison [file 12905_2022_1971_MOESM3_ESM.pdf]

**Supplementary Fig 3**  
Two-factor model performance comparison

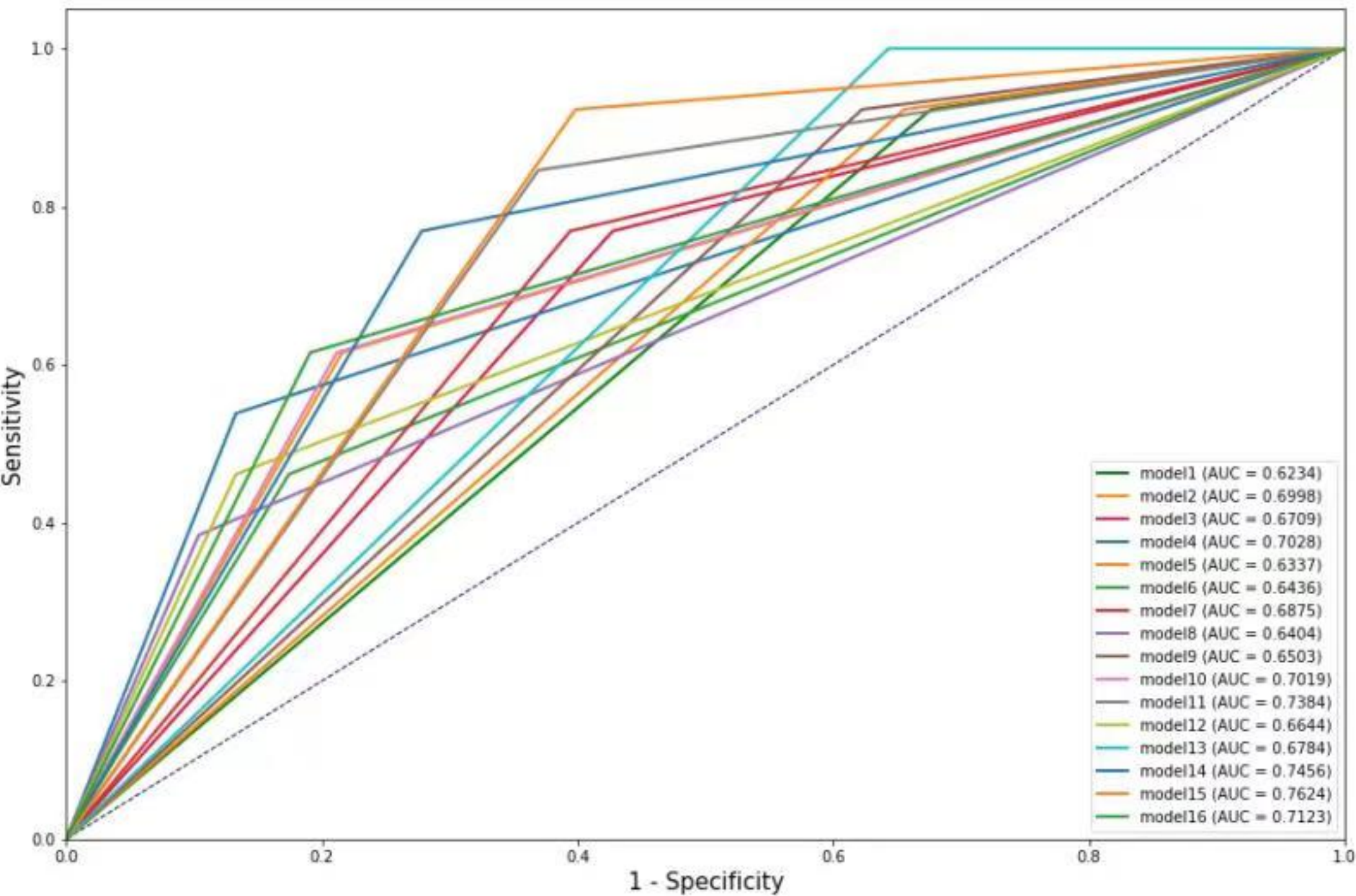

Supplement: Supplementary file 4 — Additional file 4: Fig. S3. Two-factor model performance comparison [file 12905_2022_1971_MOESM4_ESM.pdf]
